# Virus-induced Gene Silencing (VIGS) in *Nicotiana benthamiana* and Tomato

**DOI:** 10.3791/1292

**Published:** 2009-06-10

**Authors:** André C. Velásquez, Suma Chakravarthy, Gregory B. Martin

**Affiliations:** Plant Pathology and Plant-Microbe Biology, Cornell University; Boyce Thompson Institute for Plant Research

## Abstract

RNA interference (RNAi) is a highly specific gene-silencing phenomenon triggered by dsRNA^1^. This silencing mechanism uses two major classes of RNA regulators: microRNAs, which are produced from non-protein coding genes and short interfering RNAs (siRNAs). Plants use RNAi to control transposons and to exert tight control over developmental processes such as flower organ formation and leaf development^2,3,4^. Plants also use RNAi to defend themselves against infection by viruses. Consequently, many viruses have evolved suppressors of gene silencing to allow their successful colonization of their host^5^.

Virus-induced gene silencing (VIGS) is a method that takes advantage of the plant RNAi-mediated antiviral defense mechanism. In plants infected with unmodified viruses the mechanism is specifically targeted against the viral genome. However, with virus vectors carrying sequences derived from host genes, the process can be additionally targeted against the corresponding host mRNAs. VIGS has been adapted for high-throughput functional genomics in plants by using the plant pathogen *Agrobacterium tumefaciens* to deliver, via its Ti plasmid, a recombinant virus carrying the entire or part of the gene sequence targeted for silencing. Systemic virus spread and the endogenous plant RNAi machinery take care of the rest. dsRNAs corresponding to the target gene are produced and then cleaved by the ribonuclease Dicer into siRNAs of 21 to 24 nucleotides in length. These siRNAs ultimately guide the RNA-induced silencing complex (RISC) to degrade the target transcript^2^.

Different vectors have been employed in VIGS and one of the most frequently used is based on tobacco rattle virus (TRV). TRV is a bipartite virus and, as such, two different *A. tumefaciens* strains are used for VIGS. One carries pTRV1, which encodes the replication and movement viral functions while the other, pTRV2, harbors the coat protein and the sequence used for VIGS^6,7^. Inoculation of *Nicotiana benthamiana *and tomato seedlings with a mixture of both strains results in gene silencing. Silencing of the endogenous *phytoene desaturase* (*PDS*) gene, which causes photobleaching, is used as a control for VIGS efficiency. It should be noted, however, that silencing in tomato is usually less efficient than in *N. benthamiana*. RNA transcript abundance of the gene of interest should always be measured to ensure that the target gene has efficiently been down-regulated. Nevertheless, heterologous gene sequences from *N. benthamiana* can be used to silence their respective orthologs in tomato and vice versa^8^.

**Figure:**
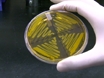


## Protocol

### Part 1: Plant material

*N. benthamiana *plants used for silencing should be around 2 ½ weeks old which is the time when the cotyledons and the first 2 - 4 true leaves have emerged. Tomato (*Solanum lycopersicum*) plants are used 7 - 8 days post emergence, when the true leaves have not yet appeared.

### Part 2: VIGS

#### DAY 1

For each experiment, *Agrobacteriumtumefaciens* harboring pTRV1, pTRV2, pTRV2-*PDS* and pTRV2-host target gene are grown on LB agar plates supplemented with 50 μg/mL of kanamycin and 100 μg/mL of rifampicin. The kanamycin selects for the pTRV plasmid while the rifampicin does so for the *Agrobacterium*. Incubate the plate at 30° C for 2 days. Silencing of PDS will cause the plants to photobleach and is used as a silencing efficiency control. Also, the genes to be downregulated must be cloned into a pTRV2 vector. There is a Gateway compatible pTRV2 vector which may facilitate the cloning and is described by Liu et al. (2002).

#### DAY 3

Inoculate a 2 - 3 mL liquid culture of LB with the above mentioned antibiotics for each of the strains. Incubate by shaking at 30 °C for 16 - 18 hours and 200 r.p.m.

#### DAY 4

Inoculate a 1 : 25 dilution of the primary culture into a secondary liquid Induction Media (IM) IM culture with kanamycin, rifampicin and 200 μM acetosyringone (Tables 1, 2 and 3). The acetosyringone is used as an inducer of the *vir* genes of *Agrobacterium *which are required for T-DNA transfer into the plant^9^ while the IM mimics the environment this pathogen encounters in the host apoplast. Incubate by shaking at 30 °C for 20 – 24 hours and 200 r.p.m.

#### DAY 5

Harvest the cells by centrifugating for 10 minutes at 3000 x g. Resuspend in the same volume that the original culture had with 10 mM MgCl_2_, 10 mM MES pH 5.5. Cells may be gently vortexed to resuspend them.
Centrifugue the cells again for 10 minutes at 3000 x g. Resuspend in half the volume of the original culture with 10 mM MgCl2, 10 mM MES pH 5.5.
Prepare a bacterial suspension with an O.D._600_ of 0.3 for each bacterial culture. Add acetosyringone to a final concentration of 400 μM to the pTRV1 culture.
Mix the cultures containing the pTRV1 and the pTRV2 (or the pTRV2 containing the gene of interest) in a 1 to 1 ratio. Also include a pTRV2-PDS control. Please note that the final acetosyringone concentration is now 200 μM and that each culture is at an O.D._600_ of 0.15.
Label the seedlings to be infiltrated with the gene to be silenced and the date of the experiment.
Poke a hole into each leaf to be infiltrated with a needle. Use a 1 mL needless syringe to infiltrate the bacterial suspension into the seedlings. For tomato, infiltrate both cotyledons while for *N. benthamiana*, infiltrate the biggest two true leaves. Five mL of each bacterial mixture should be enough to infiltrate 15 *N. benthamiana *and 25 tomato seedlings. Avoid cross-contamination by changing gloves between infiltrations and by not watering the plants until the next day after the inoculation.
The plants are kept at 20 - 22 °C in a growth chamber with a 16 hour day length and 50 % RH for at least 3 ½ weeks before they may be used for assays.

### Part 3: Representative results

Figure 1 shows a representative experiment with *N. benthamiana* and tomato plants silenced for *PDS*. Plants show the characteristic photobleaching phenotype observed in plants with diminished amounts of carotenoids. For the *PDS*-silenced control plants, photobleaching starts to be seen as soon as 1 ½ weeks after infiltration.


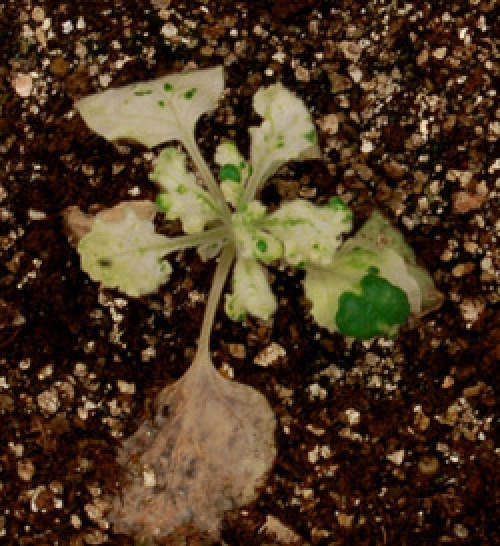
 
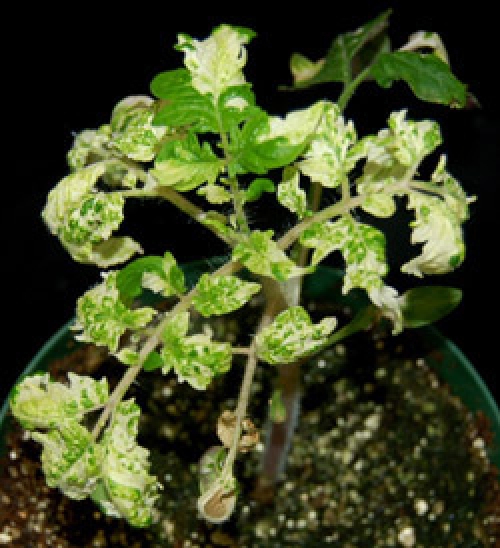


**Figure 1.** Silencing of the PDS control gene causes photobleaching in N. benthamiana (A) and tomato (B) plants. Photographs were taken 3 ½ weeks after silencing.

**Table 1.** Preparation of Induction Medium (IM).

**Table:** 

400 mL	distilled H_2_O
4.88 g	MES (2-(4 morpholino)-ethane sulfonic acid)
2.5 g	Glucose
0.12 g	NaH_2_PO_4_

Bring to a final volume of 475 mL with dH_2_O and adjust the pH to 5.6. Autoclave. After the medium has cooled down, add 25 mL of 20X AB salts.

**Table 2. **Preparation of AB salts.

**Table:** 

20 g	NH_4_Cl
6 g	MgSO_4_·7H_2_O
3 g	KCl
0.2 g	CaCl_2_
0.05 g	FeSO_4_·7H_2_O

Bring to a final volume of 1 liter with distilled water. Autoclave. Be aware that AB salts precipitate as an orange powder. Just mix them well by swirling before their use.

**Table 3.** Preparation of 200 mM Acetosyringone. Please note that acetosyringone should be prepared the day it will be used.

**Table:** 

19.6 mg	Acetosyringone (3’, 5’-Dimethoxy-4’-hydroxyacetophenone)
500 μL	DMSO (Dimethyl sulfoxide)

## Discussion

Virus-induced gene silencing is a method that allows rapid reverse genetic screens. It avoids the generation of T-DNA or transposon mediated gene knock-outs, which are only available in certain plants like *Arabidopsis* and maize. It also circumvents the time consuming process of plant transformation and allows the targeting of multiple genes at the same time, provided that they have sufficient homology^10^ or that different host target sequences are arranged in tandem in the silencing vector^6, 11^.

However, silencing is never 100% efficient and therefore, care must be taken when interpreting results. A negative result may simply indicate that the residual protein concentration was sufficient to carry out its function without obvious phenotypic consequences. Also, some constructs are better at silencing than others so it is always advisable to use at least two different mRNA regions to generate the silencing constructs for each gene. Furthermore, there is always the possibility of off-target silencing if there is sufficient homology of a gene with your silencing construct or if secondary siRNAs, which may cause transitive silencing, are produced^12^. This holds especially true for gene families. It is therefore of utmost importance to quantify the silencing efficiency of your target and off-target genes, either by RT-PCR or Northern blot analyses. If RT-PCR is chosen as the method to estimate VIGS efficiency, one of the primers should anneal to the gene outside the region targeted for silencing so that the transcript being produced by the virus is not also amplified and the results truly reflect the dowregulation of a particular endogenous gene.

VIGS efficiency is always greater in *N. benthamiana* than it is in tomato. Therefore, caution must be taken when silencing tomato seedlings. In tomato, it is critical to choose the right plant developmental stage and to maintain proper environmental conditions for viral spread. Also, gene transcript abundance must be performed for each plant under study. In addition, usually in tomato VIGS, the *A. tumefaciens *strain is GV3101 while in *N. benthamiana* either GV3101 or GV2260 are used^6,13^. It is possible to silence a gene employing a heterologous sequence from another species, provided there is sufficient homology between the two. Also, when constructing a pTRV2-target gene vector, insert lengths should be in the range of 200 to 1000 bp and they should not include homopolymeric regions (e.g. poly A tails)^14^.

If the cultures carrying pTRV2 with the genes of interest become contaminated with *PDS*, sometimes no photobleaching will be observed. Instead, plants will have a shorter stature. Therefore, it is critical to minimize any sources of cross contamination during the experiment which could potentially bias your results.
